# Using item response theory with health system data to identify latent groups of patients with multiple health conditions

**DOI:** 10.1371/journal.pone.0206915

**Published:** 2018-11-26

**Authors:** Katherine M. Prenovost, Stephan D. Fihn, Matthew L. Maciejewski, Karin Nelson, Sandeep Vijan, Ann-Marie Rosland

**Affiliations:** 1 Department of Psychiatry, University of Michigan Medical School, Ann Arbor, Michigan, United States of America; 2 Department of Internal Medicine, University of Washington, Seattle, Washington, United States of America; 3 VA Durham Center for Health Services Research and Development in Primary Care, Department of Veterans Affairs, Durham, North Carolina, United States of America; 4 School of Medicine, Duke University, Durham, North Carolina, United States of America; 5 VA Puget Sound Center of Innovation for Veteran-Centered and Value-Driven Care, Department of Veterans Affairs, Seattle, Washington, United States of America; 6 School of Medicine, University of Washington, Seattle, Washington, United States of America; 7 VA Ann Arbor Center for Clinical Management Research, Department of Veterans Affairs, Ann Arbor, Michigan, United States of America; 8 Department of Internal Medicine, University of Michigan, Ann Arbor, Michigan, United States of America; 9 VA Pittsburgh Center for Health Equity Research and Promotion, Department of Veterans Affairs, Pittsburgh, Pennsylvania, Unites States of America; 10 Department of Internal Medicine, University of Pittsburgh, Pittsburgh, Pennsylvania, United States of America; Duke-NUS Medical School, SINGAPORE

## Abstract

A critical step toward tailoring effective interventions for heterogeneous and medically complex patients is to identify clinically meaningful subgroups on the basis of their comorbid conditions. We applied Item Response Theory (IRT), a potentially useful tool to identify clinically meaningful subgroups, to characterize phenotypes within a cohort of high-risk patients. This was a retrospective cohort study using 68,400 high-risk Veteran’s Health Administration (VHA) patients. Thirty-one physical and mental health diagnosis indicators based on ICD-9 codes from patients’ inpatient, outpatient VHA and VA-paid community care claims. Results revealed 6 distinct subgroups of high-risk patients were identified: substance use, complex mental health, complex diabetes, liver disease, cancer with cardiovascular disease, and cancer with mental health. Multinomial analyses showed that subgroups significantly differed on demographic and utilization variables which underscored the uniqueness of the groups. Using IRT models with clinical diagnoses from electronic health records permitted identification of diagnostic constellations among otherwise undifferentiated high-risk patients. Recognizing distinct patient profiles provides a framework from which insights into medical complexity of high-risk patients can be explored and effective interventions can be tailored.

## Introduction

Increasing numbers of US adults have multiple co-existing health conditions[1] and these patients account for a disproportionate share of healthcare costs.[2–4] Characterizing the multiple medical conditions and needs of this complex population has become a top priority for healthcare systems.[5–7] Traditionally, management of patients with multiple coexisting conditions has been informed by clinical guidelines targeted to individual medical conditions that do not fully account for the complex interplay among multiple conditions for individual patients.[8–10] A fundamental step towards developing effective strategies for managing complex patients is to characterize these aspects of patient heterogeneity.[6] From an analytic standpoint, two important sources of patient heterogeneity need to be considered: (a) the interactions of particular medical conditions *within* individual patients, and (b) the variation in complexity *between* patients from qualitatively different clinical subpopulations.

Prior work to understand multi-morbidity has applied model-based methodologies to stratify patient populations into subgroups based on particular combinations of medical conditions, such as classification and regression tree (CART) analysis,[11] cluster analysis,[12, 13] multiple correspondence analysis,[14] factor analysis,[12] and latent class analysis (LCA).[15–22] Latent variable models, such as LCA by itself or combined with other models, are particularly promising methods because they attempt to “unmix” complex populations by revealing underlying patterns or subgroups that may not be readily apparent, yet clinically meaningful.[23, 24] LCA assumes there are qualitatively distinct patient populations with different defining patterns.[25] Another technique that expands upon the LCA approach is mixture distribution Item Response Theory (MD-IRT). Mixture distribution IRT combines latent class analysis (LCA) with IRT. This is unique in that it, not only identifies separate groups of people, but also translates the underlying construct, for example, medical complexity, into numerical estimates of the degree of complexity represented within each medical condition, and also within each patient, for different groups of patients, which is not a feature of traditional LCA.[26–28] Further, IRT, unlike other techniques, can efficiently characterize patient complexity by requiring only a limited set of defining medical conditions[26]. MD-IRT has not, to our knowledge, been used with data from electronic health records to better understand comorbidity distributions among complex patients. Here, we define complex patients as having several chronic conditions. We sought to understand if the application of MD-IRT could help identify subgroups of patients based on constellations of medical conditions, and quantify medical complexity of salient medical conditions for each subgroup (i.e., medical conditions essential for characterizing subgroups that represent all levels of the complexity continuum). We postulated that MD-IRT models could establish clinically meaningful subgroups among otherwise undifferentiated medically complex patients. Identifying subgroups within a population of high-risk patients could potentially inform clinical management.

## Methods

### Sample

The data were not fully anonymized nor did they provide written consent. This study was deemed non-research, and the requirement for IRB approval was waived. Dr. Angela Denietolis, Executive Director of the Office of Primary Care, designated the study as non-research. VHA Program Office Officials are authorized under VHA Handbook 1058.05 to document the status of non-research operations activities that are funded, mandated, managed, sponsored, or otherwise supported by the Official’s Program Office. The study sample included adult patients receiving care from the Veteran’s Health Administration (VHA) in 2014 that were at high-risk for hospitalization. Risk of hospitalization was defined using the Care Assessment Needs Score (CAN-2-H), that was developed in VA to predict the probability of hospitalization during the next year based on demographic characteristics (e.g., age, sex, military service information), clinical information (e.g., medical conditions, vital signs, medications and laboratory tests) and administrative data (e.g., use of health services) extracted from VHA administrative files, and US census data.[29] A CAN-2-H score is computed weekly for Veterans who have been seen by a VA primary care provider within the past 2 years, are living, and are not currently hospitalized. Patients with a CAN-2-H score in the 90^th^ percentile (i.e., those with a high risk of hospitalization that was greater than 90% of the patient population) at any time during the VA fiscal year (FY) 2014 were initially selected (N = 697,635) because these patients are flagged for attention by VA primary care teams. Patients in this percentile had a predicted risk of hospitalization, within the year, of 25% or more. Patients were omitted if their medical record had missing information on the presence *or* absence of at least 131 (90%) of the initially examined 145 medical conditions described in the ‘Measures’ section below (n = 5,888 patients; <1% of the sample initially examined). Patients were also excluded if they were missing data on whether they were screened for PTSD (n = 6,451; 1%), since the medical condition of PTSD was deemed potentially important for Veterans. Finally, patients who had none of the 31 medical conditions included in the analyses (n = 1,129) were excluded yielding a final eligible pool of 684,004. Due to the intensive computational requirements, a 10% random sample of 68,400 Veterans from this pool was analyzed (Table 1).

**Table 1:** Study sample characteristics (N = 68,400).

**Patient characteristics**	**%Prevalence**
Age ≥65	56%
White, non-Latino^1^	59%
Married	37%
Unemployed	6%
≥5 Outpatient visits in 90 days	55%
>15 Non-face to face encounters-	79%
(per year)	
≥3 ED visits per year	41%
≥1 Inpatient admission per year	62%
**Diagnoses**	**%Prevalence**
Depression	40%
Anxiety	19%
Post-traumatic stress disorder	22%
Serious mental illness^2^	24%
Bipolar	7%
Psychosis	9%
Drug abuse	20%
Alcohol abuse	18%
Nicotine abuse	29%
Hypertension	72%
Coronary artery disease	29%
Congestive heart failure	21%
Cardiac arrhythmias	24%
Chronic pulmonary disease	33%
Cerebrovascular Disease	13%
Peripheral vascular disease	17%
Clotting disorders	6%
Diabetes	43%
Electrolyte disorders	15%
Thyroid disorders	11%
Chronic renal failure	17%
Acute renal failure	10%
Polyneuropathy	12%
Liver disease	13%
Chronic hepatitis	10%
Chronic arthritis	47%
Chronic pain	77%
Weight loss	7%
Anemia	20%
Malignant tumor-	21%
(Cancer including recurrence)	
Malignant neoplasm-	22%
(non-relapse cancers)	

^1^White vs. non-white or undisclosed

^2^Includes episodic (bipolar conditions) and unspecified paranoid disorders. (12.5% undisclosed).

### Measures

Medical conditions were defined from ICD-9 codes in patients’ inpatient and outpatient VHA and VA-paid community care encounters as recorded in the administrative VHA electronic health records database. A clinical rater (AMR) reviewed ICD-9 codes commonly used to define medical conditions in other comorbidity scales[30] and selected codes limited to those reflecting chronic conditions relevant to primary care. Of the 145 initially examined medical conditions, 85 conditions with prevalence <5% were eliminated because they lacked sufficient variability for analysis (e.g. 1.2% of the 684,004 patients had HIV, 4.3% had epilepsy, 2.7% had osteoporosis, and 1.6% had Parkinson’s Disease). Twenty-nine overlapping conditions were eliminated (i.e., common ICD-9 definitions used in the EHR and those from the clinical rater), for a final set of 31 chronic conditions (Table 1 shows prevalence rates of the 31 conditions; see S1 Table for ICD-9 definitions and chronic conditions used). Other variables of interest included age (> = 65 years old vs <65 years old), sex, race (white vs other/undisclosed; 12.5% were undisclosed), marital status (married vs unmarried/unknown; 0.8% unknown), and unemployment status were obtained from VA administrative data (Table 1). Four types of health care service indicators, based on VA outpatient encounter and VA inpatient admission data, were constructed: number of VA outpatient office visits (≥5 in 90 days); number of non-face-to-face VA encounters, e.g., phone encounters (≥15 in a year); VA emergency department visits (≥3 in a year); and VA hospital admissions (≥1 in a year).

### Analyses

**Fig 1:**
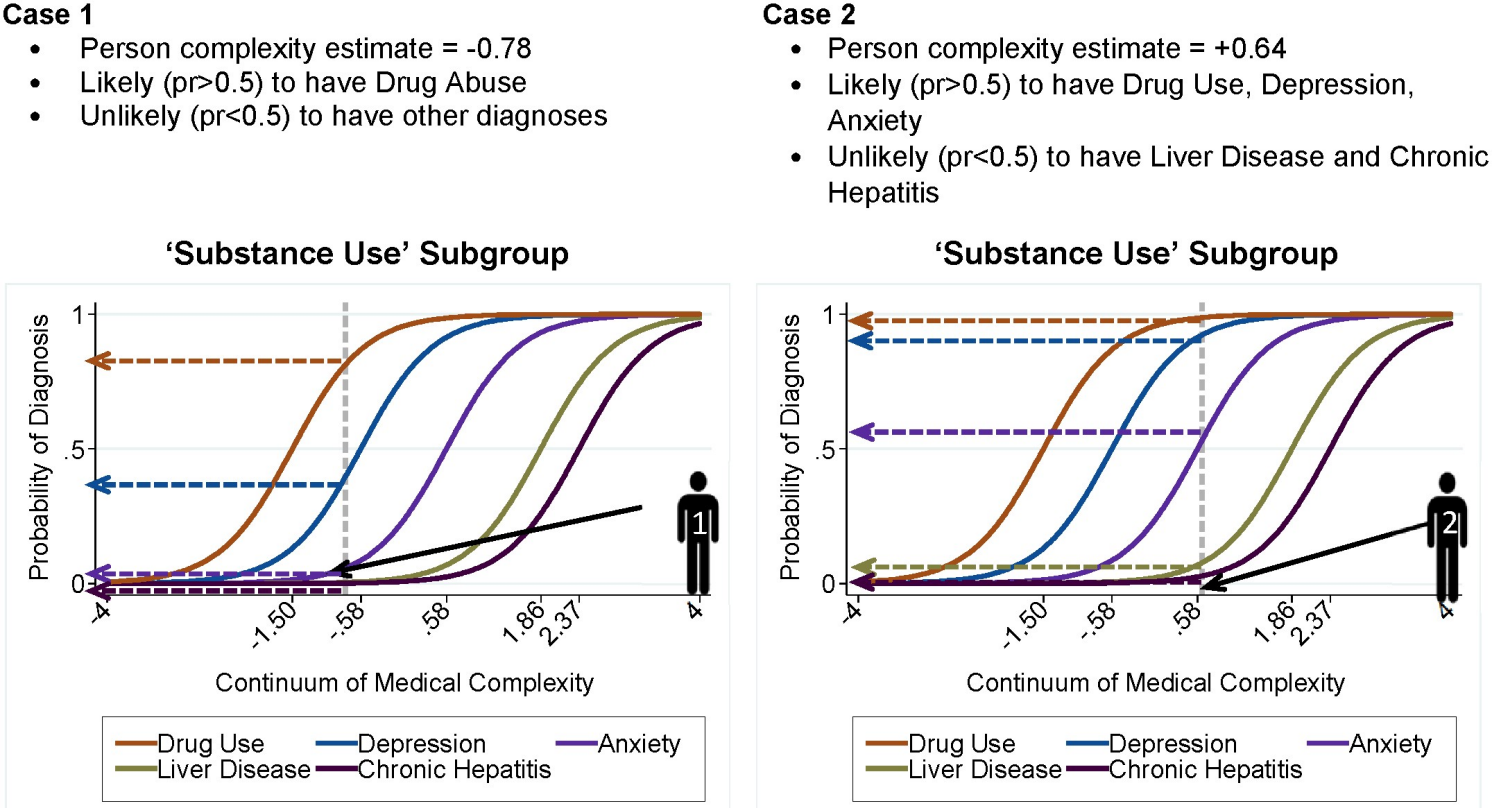
Interpreting IRT item characteristic curves (ICC) as defined by medical diagnoses for two example patients from the same subgroup. This figure illustrates fundamental aspects of IRT models using two hypothetical patients from the same subgroup. Medical conditions and persons are each given estimates which place them on the construct, medical complexity; estimates represent amount of complexity captured by the medical condition or person. Here, the two patients represent low and high amounts of complexity and consequently, which medical conditions they were likely to have given their levels of complexity.

The following is a brief explanation about the models used. If the reader prefers a more detailed explanation, please see the section, ‘Technical details’ and the end of the Methods section. Item Response Theory models ordinarily use ‘correct’ or ‘incorrect’ responses to questions that represent a latent construct, for example, “verbal reasoning” in a standardized achievement test. In this study, we used the presence or absence of medical conditions to model the latent construct, “medical complexity”. Specifically, mixture distribution IRT was used to identify patient subgroups while estimating both how much medical complexity each diagnosis represents for a subgroup and patient complexity within groups. Estimates for medical condition and patient medical complexity are quantified to be on the same metric, in this case increasing amount of complexity (Fig 1; Technical details, section ‘Mixture Distribution IRT (MD-IRT)’. Fig 1 uses item characteristic curves (ICCs) to illustrate the concept that these models place persons and medical conditions on the *continuum of medical complexity*. The *x*-axis represents the complexity continuum on an interval scale, the *y*-axis is the probability that a person would have a particular medical condition given her/his degree of complexity, and the curves display the nonlinear relationship between the two. Increasing values on the *x*-axis indicate increasing levels of complexity a person would likely have if s/he were to have *that* condition in be in *that* subgroup; medical conditions indicating compounding complexity are further to the right. The location of a medical condition on the construct (often expressed as “difficulty” in IRT) is the value on the continuum at which a person with that level of complexity would be just as likely to have or not have (probability = 0.5) that medical condition.

To examine whether medical complexity could be better understood by identifying subpopulations of patients with different coexisting chronic conditions, including patient-level subgroup-specific complexity, we constructed mixture distribution IRT models for medical condition indicators, examining 1 to 7 latent classes using mixRasch, version 1.1 in R release 3.3.0.[31] Because the optimal model for clinical application is not solely a statistical issue,[32] the optimal number of classes (or ‘subgroups’) was determined by the fit index, Bayesian Information Criterion (BIC) where lower values indicate better fit, and clinical interpretability.[23, 33, 34] Subgroups were defined by distinct patterns of conditions and patients were assigned to the subgroup with the combination of medical conditions that most resembled their own. Each patient was assigned to only one subgroup resulting in mutually exclusive groups.

### Subgroup-specific models

To meet our goals of efficiency (reducing the 31 medical conditions to only the most relevant for each class) and flexibility (by inviting the possibility of fitting IRT models defined by different medical conditions for each subgroup), final assessments of assumptions and person fit were made separately by subgroup.

Since the intent of this study was to present parsimonious models with straightforward interpretation, a restrictive set of scaling analyses were used to identify only the most representative, unidimensional, group-specific, medical conditions. After subgroups were identified, Mokken scaling[35–38] was employed within each subgroup to retain only those diagnoses that satisfied IRT assumptions, as well as identify the patients that fit the model well (i.e., were appropriately described by the model; Technical details, section: ‘Subgroup-specific models and assumptions’ provides more detail on Mokken scaling and assumptions). Mokken scaling is an extension of the Guttman structure which stipulates that, in this case, medical conditions are cumulative on the complexity construct continuum and reflect greater levels of complexity as conditions are located at the higher end of the continuum of medical complexity (Fig 1). Guttman errors for patients were used to identify those patients with inconsistent medical condition patterns.[36, 37] For example, if in a particular subgroup, having hypertension (HTN) represents less complexity than congestive heart failure (CHF), then a person in that group diagnosed with CHF will likely be diagnosed with HTN as well. A person, who had a diagnosis of CHF but not HTN, would be an example of a Guttman error. A certain degree of error is tolerated in the models in that people with inconsistent patterns are still included. Consistent with our goal of presenting a clear interpretation of the concepts, patients with more than one Guttman error were categorized as ill-fitting and excluded.

There are different forms of the IRT model. An IRT 1PL (one parameter logistic) model estimates one parameter that yields a common slope (“item discrimination”) for each item characteristic curve (Fig 1) indicating how well the medical conditions overall represent the construct. An IRT 2PL model allows each diagnosis to have different discrimination estimates (i.e., each diagnosis has its own slope). IRT 1PL and 2PL models were run for each subgroup separately only including the resultant diagnoses from the Mokken scaling, thus fulfilling the assumptions, and using patients that had good model fit (Technical details, sections: ‘Subgroup-specific models and assumptions’ and ‘Person fit’).

Characterization of subgroups was based on salient medical conditions identified by the IRT models, and other highly prevalent medical conditions not able to be used in the models. This was then augmented with multinomial logistic regression models predicting class membership from utilization and demographic data. A Scheffé correction was used to adjust for multiple comparisons.[39] These analyses were performed in Stata/MP release 14 (StataCorp LP, College Station, TX).

### Technical details

#### Mixture distribution IRT (MD-IRT)

Item response theory (IRT) models are a class of measurement models used widely in the fields of education and psychology to identify subgroups because they are designed to simultaneously quantify people and qualities of responses to a challenge, such as a test question (or, in this case, a medical diagnosis), in relation to a latent process (construct).[26, 27] In the case of patterns of medical comorbidities, the construct of interest, “medical complexity”, is defined as the co-occurrence of certain medical conditions, which create a medically complex patient profile. IRT models can characterize this complexity efficiently—successful characterization of a person’s complexity can be made with a limited set of key defining medical conditions[26] that MD-IRT translates into numerical estimates—quantifying both the indicator of the construct and person’s level of the construct. That is, in our case, these values represent the degree of complexity embodied in a medical condition or patient in a particular subpopulation.

Mixture Distribution IRT (MD-IRT) models were used as a starting point to identify latent classes. The basic form of this model can be expressed as[40]

$$p\left(x|\theta ,c\right)=\overset{I}{\underset{i=1}{\Pi }}\frac{exp\left[x_{i}\left(\theta -\beta_{ic}\right)\right]}{1+exp\left(\theta -\beta_{ic}\right)}$$ (1)

where:

*x* Medical condition vector of person (binary: 1 = has medical condition; 0 = does not have)

*θ* “Medical complexity” estimate for person

*c* Latent class *c*

*x*_*i*_ Person’s status (1 vs 0) on medical condition *i*

*β*_*ic*_ Location (“difficulty”) of medical condition *i* on the construct, complexity, in class *c*

This defines the conditional probability that a person would have a set of medical conditions given his/her estimated medical complexity (*θ*) and membership in class *c*. The right side expresses the probability of a person’s medical condition as a function of the difference between the locations of person and medical condition on the construct continuum (complexity). If, for example, the individual and medical condition represent the same amount of complexity, i.e., have the same estimated locations, the probability of having that medical condition is 0.5. This translates to estimating the probability of a person having a particular diagnosis while considering her/his level of medical complexity, which subgroup they are in, and how much complexity that particular diagnosis represents for her subgroup. Because latent classes represent qualitatively different subpopulations, medical conditions represent medical complexity *differently* for each class; the *β* estimates for a specific medical condition can differ among classes. To assign a person a particular class, the model estimates probabilities of each person’s diagnostic pattern occurring in each class (each with its own characteristic medical condition pattern). In this study, all patients were assigned to the subgroup that most closely matched their own pattern of medical conditions, i.e., the group associated with the highest probability.

#### Model fit index: Bayesian Information Criterion (BIC)

The Bayesian Information Criterion (BIC) index indicates model fit by adjusting models’ log likelihood estimates for number of parameters estimated and sample size; lower relative values are considered “better” model fits regardless of nesting.[23, 33, 34]

#### Subgroup-specific models and assumptions

Assumptions, dimensionality, and appropriateness of the model for each person (person fit) were assessed within each latent group separately. Though all patients were used in the MD-IRT models, final assessments of assumptions and person fit were assessed separately within the subgroups. This was done with the goal of efficiency (reducing the 31 diagnoses to only the most relevant for each class) and flexibility by inviting the possibility of fitting different IRT models (Rasch; 1PL; 2PL) for each subgroup. In IRT, 1PL refers to a model where one parameter is estimated that yields a location estimate for each diagnosis on the construct continuum and how well each diagnosis represents the construct and “item discrimination” (parameter defining how well a particular condition identifies persons’ location of the construct) is set to a constant; Rasch is a 1PL model where item discrimination is set to a value of 1.7. Though simplicity was a priority, 2PL IRT models were considered based upon not only the estimated location of each diagnosis on the underlying continuum, but on different estimates of how well each diagnosis was measuring the underlying construct.

After subgroups were established, skewness was assessed in each subgroup by examining diagnosis prevalence; any condition with <5% or >95% prevalence was omitted for that subgroup. Although diagnoses with >95% prevalence were not included in the subgroup models, they were later used descriptively to help describe the nature of the groups. The IRT assumptions of unidimensionality, monotonicity, and local independence were verified, assumptions and medical conditions were selected for each subgroup using Monotone Homogeneity (MH) Mokken scaling, an extension of Guttman scaling, and creates data that meet the IRT assumptions of unidimensionality, monotonicity, and local independence.[35–38] Within each subgroup, trace lines were examined to identify conditions that violated monotonicity, i.e., the probability of having a diagnosis increases as number of conditions a person has (representing greater amounts of the underlying construct) increases. Unidimensionality implies that all diagnoses (within a subgroup) are explained by one common latent construct and local independence stipulates that all interrelationships between diagnoses are explained by the underlying construct, i.e., there is no residual correlation among diagnoses after accounting for the model. These assumptions are related. If local independence holds, then the underlying construct is sufficient to explain the data, and it is assumed to be a unidimensional construct. Loevinger’s H coefficients of diagnoses were used to verify fit to a Mokken scale structure which ensures these assumptions are met.[35, 36] These, and all subsequent analyses, were performed in Stata/MP release 14 (StataCorp LP, College Station, TX).

#### Person fit

The number of Guttman errors, as measured by the Loevinger’s H coefficient, was used to identify persons with inconsistent diagnosis patterns.[36, 37] Patients with more than one error were categorized as ill-fitting (i.e., the framework of the model failed to describe the person’s data adequately) and were excluded. A Guttman structure stipulates that medical conditions are cumulative on the complexity construct continuum and reflect greater levels of complexity as conditions are located at the higher end of the continuum (Fig 1). A Guttman error would occur if a person had CHF and HTN but not diabetes. To promote a clear interpretation of the concepts, patients with more than one Guttman error were categorized as ill-fitting and excluded from further analyses.

## Results

The initial MD-IRT models were run on 29 of the 31 diagnoses due to consistently poor fit for two conditions—serious mental illness and both cancer measures. Malignant neoplasm was chosen, although both were used to describe subgroups. Given model fit indices (Table 2) and clinical interpretability, a 6-class model was selected as it had a relatively low BIC (1,618,830) with good clinical interpretability. The 7-class solution had the lowest BIC (1,613,357) but produced a subgroup that overlapped substantially with two other groups (Cardiac+Cancer and Cancer+Mental Health) and discarded. Assumptions of unidimensionality, monotonicity, and local independence were satisfied for each subgroup based on results of the Mokken analyses. Across all subgroups, a Guttman structure appropriately described cumulative medical condition patterns for 85% of the sample (N = 62,579) which then became the final sample. Both IRT 1PL and 2PL models were run within each subgroup using only the salient conditions defined by Mokken analyses.

**Table 2:** Fit indices of mixture distribution IRT models: 1–7 class solution based on 29 medical conditions (N = 68,400 patients).

#Latent classes	-2 Log Likelihood	#Parameters estimated	BIC
1	1,769,508	50	1,769,704
2	1,681,100	101	1,682,223
3	1,658,348	152	1,660,039
4	1,643,788	203	1,646,048
5	1,621,948	254	1,624,776
6	1,615,130	305	1,618,830
7	1,609,394	356	1,613,357

**Fig 2:**
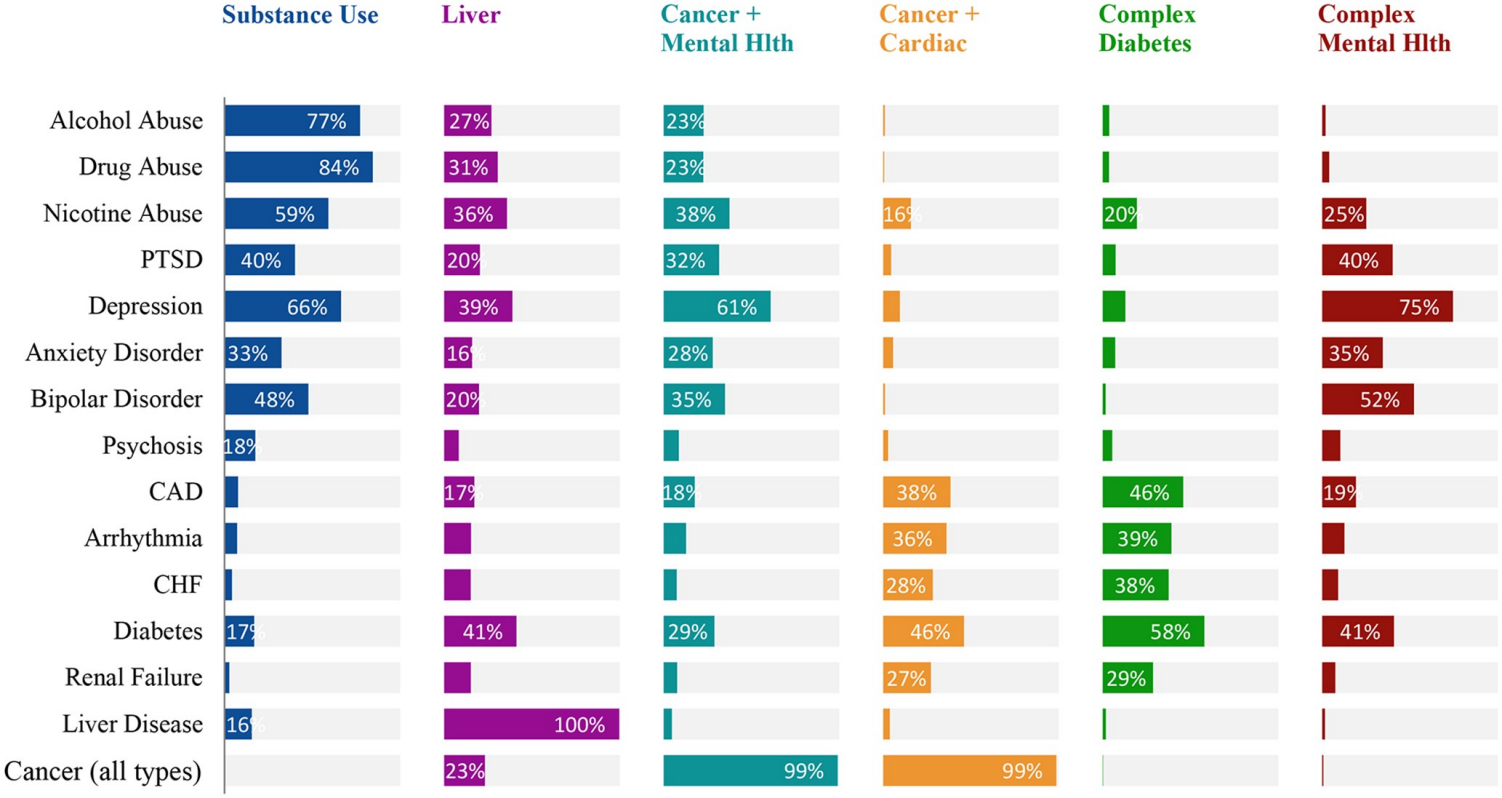
Prevalence of medical conditions by subgroup (prevalence rates <15% not labeled). This bar chart shows the prevalence of selected medical condition used to help label the subgroups. Not all of these medical conditions were included in the final models because they either were too prevalent (> 95%) or not prevalent enough (< 5%), but were presented here descriptively to help identify the nature of the groups.

**Fig 3:**
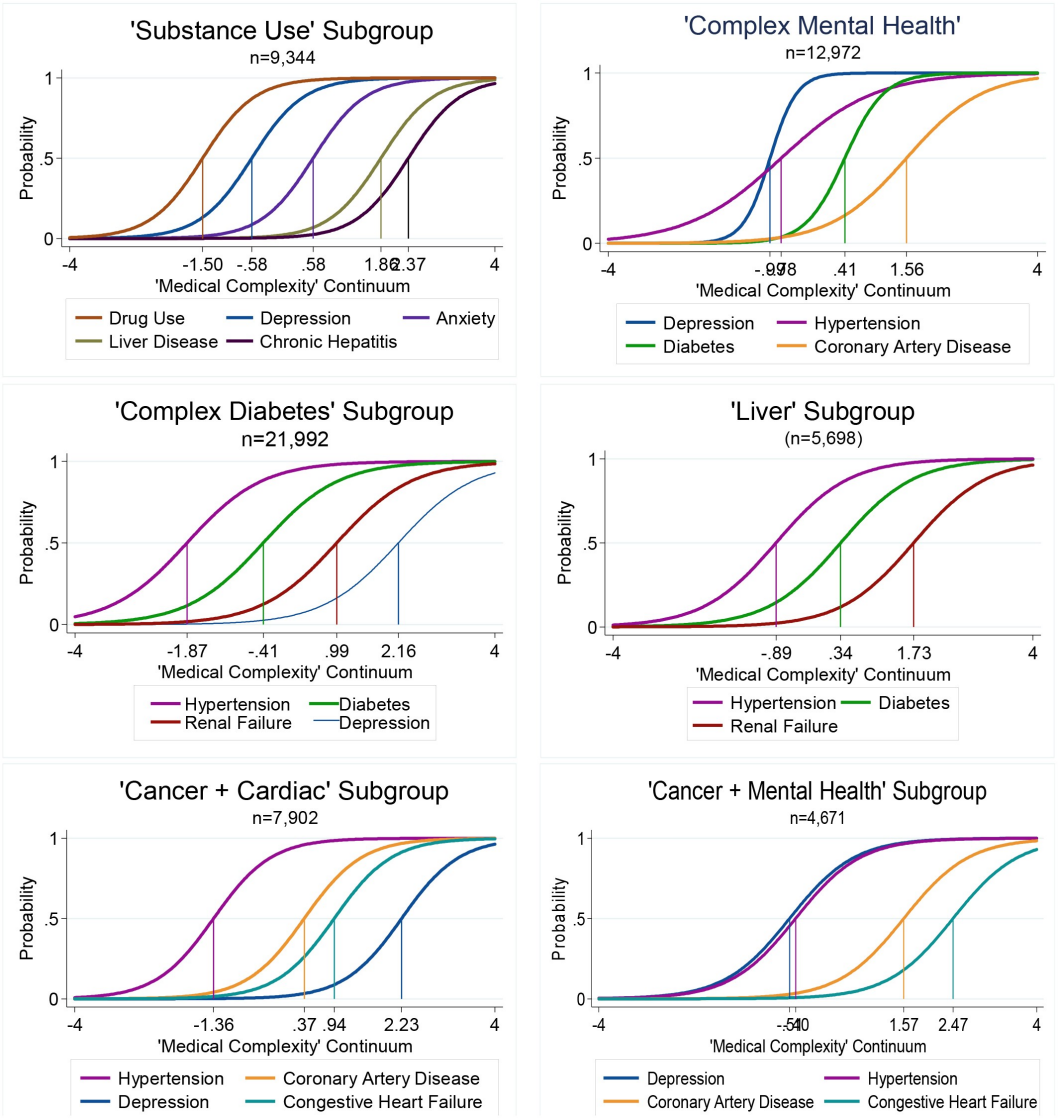
Item characteristic curves showing probability of having a medical condition as a function of amount of medical complexity and patient subgroup. As previously demonstrated in Fig 1; a medical condition in one group may reside at the lower end of the complexity continuum, while in another group, the same condition represents extreme complexity.

IRT 2PL models were not required for most subgroups (Cardiac+Cancer, Complexity Diabetes, Substance Use, Liver Disease, and Cancer+Mental Health) because separate slopes per condition were similar enough to justify the more parsimonious 1PL models (see S2 Table for model coefficients). The complex Mental Health group was the only one for which a 2PL model was most appropriate. Fig 2 shows the prevalence of various medical conditions by subgroup, and Fig 3 displays ICC curves of salient medical conditions with the estimated location of each medical condition on the underlying complexity continuum for each subgroup.

### Subgroup identification

#### Subgroup 1: “Substance Use”

The final subgroup-specific IRT 1PL model fit well (i.e., having a pattern of comorbidities consistent with the characteristic pattern of this group) for 88% (n = 9,344) of the “Substance Use” subgroup (based on fewer than two Guttman errors), and 97% (n = 9,041) of these individuals were diagnosed with drug abuse (n = 8,176; 88%) and/or alcohol abuse (n = 7,157; 77%). Twelve percent (n = 1,235) of the patients originally assigned to this group, based on the MD-IRT model, were not described well by this subgroup’s model. Alcohol abuse did not fulfill the monotonicity assumption and was excluded from the final IRT model. Increasing complexity was indicated by the additional presence of five medical conditions: drug abuse, depression, anxiety, liver disease, and chronic hepatitis (Fig 3) while a patient with only drug use or depression would not be as medically complex *for this group*.

#### Subgroup 2: “Complex Mental Health”

The IRT 2PL model fit well for 89% of patients in this subgroup (n = 12,972 out of the 14,649 patients initially assigned) and included four conditions. Most patients in this group had a diagnosis of depression (10,544 of 12,972; 81%). Based on steeper slopes, depression and diabetes were particularly representative of this group (Fig 3). Depression and hypertension were the common comorbidities representing lower levels of complexity for this group. Diabetes reflected increased complexity and the addition of coronary artery disease is particularly indicative of high complexity for this group (Fig 3).

#### Subgroup 3: “Complex Diabetes”

The IRT 1PL model fit well for 93% of patients in this group (n = 21,992 of 23,691 in the original group). Salient medical conditions in order of complexity include hypertension, diabetes, renal failure, and depression. In this subgroup, depression was an important marker of a very complex medical profile. For example, patients in this group, with diabetes and depression had much more medical complexity than a person with depression alone.

#### Subgroup 4: “Liver Disease”

The IRT 1PL model fit well for 98% of patients in this group (n = 5,698 of 5,826). Virtually every member of this class had liver disease (n = 5,682 of 5,698; 99.7%) or chronic hepatitis (n = 5,410; 95%; see S1 Table 1 for distinctions between these conditions); hence, liver disease was almost universally necessary for classification of this group. Intermediate complexity was marked by hypertension or diabetes, while a diagnosis of renal failure indicated high accumulated complexity (Fig 2).

#### Subgroup 5: “Cancer with Cardiovascular Disease”

The cancer diagnosis was associated with two subgroups. One, for which an IRT 1PL model fit well for 92% of the patients (n = 7,902 of 8,629), included not only a diagnosis of malignant cancer in 97% (n = 7,649; and 99% when considering all cancers, n = 7,823), but also cardiovascular conditions, most commonly hypertension, and less often coronary artery disease and congestive heart failure. An added diagnosis of depression indicated with a high degree of medical complexity.

#### Subgroup 6: “Cancer with Mental Health”

The second subgroup of patients with malignancy for which an IRT 1PL model fit well for 93% of patients in this group (n = 4,671 of 5,026) where nearly all (98%) had a malignant tumor medical condition and a diagnostic pattern, reflecting increasing degrees of complexity, of cancer, depression, hypertension, coronary artery disease, and congestive heart failure. In this subgroup having depression and hypertension, in addition to malignancy, was common, hence likely found in nearly all members. Notability, depression and hypertension were located at comparable places on the construct, suggesting that either was a marker of the lesser complex patient, so only one would help characterize people.

### Characterization of subgroups: Demographic and service utilization

Despite being drawn from a group of patients at similarly high-risk for hospitalization, the 6 groups significantly differed on most utilization and demographic variables; Table 3 contains detailed descriptive information for each subgroup.). For bivariate analyses, most variables had to be dichotomized because of too few respondents in more finely divided categories. Binary indicators were defined by the most populated category versus the combination of all the other categories.

**Table 3:** Descriptive demographic and utilization information within subgroups, %(*n*).

	Latent Classes					
	Substance Use (n = 9,344)	Complex Mental Health (*n* = 12,972)	Complex Diabetes (*n* = 21,992)	Liver Disease (*n* = 5,698)	Cancer+Cardiac (*n* = 7,902)	Cancer+Mental Health (*n* = 4,671)
Marital Status						
Married	19.3% (1,802)	37.1% (4,814)	41.7% (9,180)	30.3% (1,724)	46.9% (3,703)	38.1% (1,780)
Divorced	41.4% (3,871)	35.1% (4,557)	30.4% (6,686)	39.4% (2,244)	27.6% (2,178)	35.9% (1,676)
Single	38.5% (3,600)	26.9% (3,486)	27.1% (5,967)	29.6% (1,688)	25.0% (1,973)	25.2% (1,176)
Unknown	0.8% (71)	0.9% (115)	0.7% (159)	0.7% (42)	0.6% (48)	0.8% (39)
Male sex	92.1% (8,604)	84.3% (10,940)	96.6% (21,243)	96.0% (5,469)	97.7% (7,717)	93.5% (4,365)
Age						
18–54	41.8% (3,906)	25.3% (3,279)	5.2% (1,147)	9.8% (556)	1.8% (138)	8.7% (407)
55–64	38.6% (3,606)	33.7% (4,377)	21.6% (4,747)	51.7% (2,944)	13.7% (1,080)	29.4% (1,371)
65–74	17.6% (1,641)	31.4% (4,079)	40.1% (8,807)	33.6% (1,915)	40.7% (3,219)	47.1% (2,202)
75–84	1.6% (146)	6.0% (780)	19.2% (4,228)	4.1% (231)	26.2% (2,071)	9.8% (459)
85+	0.5% (45)	3.5% (457)	13.9% (3,063)	0.9% (52)	17.6% (1,394)	5.0% (232)
Unemployed (2014)	22.2% (2,075)	5.7% (743)	1.3% (295)	6.8% (386)	0.3% (27)	4.0% (185)
Race						
White	50.9% (4,756)	59.1% (7,668)	61.8% (13,583)	48.5% (2,763)	62.3% (4,921)	59.3% (2,771)
Black	25.5% (2,384)	17.4% (2,260)	18.5% (4,076)	26.8% (1,524)	18.6% (1,472)	19.6% (914)
Hispanic	5.1% (479)	6.8% (877)	5.3% (1,167)	6.9% (394)	4.8% (378)	5.0% (233)
Multiracial	2.5% (233)	2.2% (289)	1.8% (386)	2.3% (128)	1.7% (135)	1.7% (79)
Other	1.5% (141)	1.6% (207)	1.6% (344)	1.1% (65)	1.3% (100)	1.1% (50)
Unknown	14.5% (1,351)	12.9% (1,671)	11.1% (2,436)	14.5% (824)	11.3% (896)	13.4% (624)
CAN score^1^, m(sd)	45 (.16)	.40 (.13)	.49 (.18)	.44 (.16)	.48 (.17)	.46 (.16)
#Office visits (prior 90 days)						
0 or unknown	4.4% (407)	2.1% (273)	3.3% (714)	3.3% (189)	1.6% (124)	1.5% (72)
1–2	45.3% (4,231)	39.0% (5,064)	46.7% (10,271)	40.7% (2,321)	36.2% (2,863)	32.7% (1,525)
3+	50.4% (4,706)	58.9% (7,635)	50.1% (11,007)	56.0% (3,188)	62.2% (4,915)	65.8% (3,074)
#Non-face encounters (prior year)						
< 4	8.9% (829)	4.5% (581)	4.5% (996)	4.0% (229)	2.5% (200)	3.3% (155)
4	22.0% (2,056)	17.8% (2,314)	16.7% (3,678)	14.0% (800)	10.6% (835)	14.0% (655)
5	69.1% (6,459)	77.7% (10,077)	78.8% (17,318)	81.9% (4,669)	86.9% (6,867)	82.7% (3,861)
#ER visits (prior year)						
0/no information	13.0% (1,218)	14.3% (1,852)	16.0% (3,510)	17.2% (982)	18.5% (1,462)	20.9% (974)
1–2	41.0% (3,829)	44.0% (5,712)	45.0% (9,905)	41.8% (2,383)	43.3% (3,424)	42.9% (2,005)
3+	46.0% (4,297)	41.7% (5,408)	39.0% (8,577)	40.9% (2,333)	38.2% (3,016)	36.2% (1,692)
Inpatient admission (prior year)						
0	32.4% (3,024)	46.1% (5,974)	36.3% (7,991)	35.6% (2,029)	35.1% (2,772)	43.0% (2,007)
1	67.6% (6,320)	54.0% (6,998)	63.7% (14,001)	64.4% (3,669)	64.9% (5,130)	57.0% (2,664)

^1^ Probability of hospitalization risk at 1-year; means and standard deviations are shown.

Results of the statistical tests, including the predicted probabilities of having specific characteristics in each subgroup, are presented in Table 4. “Substance Use” subgroup members’ utilization profiles were most distinct from the other groups; this group was most likely to have had ≥3 visits to a VA emergency department (pp = 0.46) and at least one VA hospitalization during the prior year (pp = 0.68). They were least likely to make use of non-face-to-face encounters (pp = 0.69) and (along with the “Complex Diabetes” group), least likely to have had ≥5 office visits during the prior 90 days (pp = 0.50). The “Cancer+Cardiac” group members most frequently used non-face-to-face encounters (pp = 0.87). The “Cancer with Mental Health” group had the greatest proportion of frequent office visits (pp = 0.66). “Substance Use” subgroup members were most likely to be unemployed (predicted probability, pp = 0.22), least likely to be married (pp = 0.19), youngest (pp = 0.20), more racially diverse (along with the “Liver” group; pp = 0.51 and pp = 0.48 respectively). The “Cancer+Cardiac” group members were most likely to be men (pp = 0.98), married (pp = 0.47), older (pp = 0.85), white race (along with the “Complex Diabetes” group; pp = 0.62 for both groups).

**Table 4:** Predicted probabilities (pp), with 95% confidence intervals, indicating the likelihood of the classes being in the listed categories of demographic and service utilization factors, based on multinomial logistic regression analyses (N = 62,579)^1^.

	Latent Classes					
	Substance Use	Complex Mental Health	Liver	Complex Diabetes	Cancer+ Cardiac	Cancer+Mental Health
Married	19 (.18, .20)	.37^***A***^ (.36, .38)	.30 (.29, .31)	.42 (.41, .43)	.47 (.46, .48)	.38^***A***^ (.37, .40)
Male sex	92^***B***^ (.92, .93)	.84 (.84, .85)	.96^***C***^ (.95, .96)	.97^***C***^ (.96, .97)	.98 (.97, .98)	.93^***B***^ (.93, .94)
Age 65+ years old	20 (.19, .20)	.41^***D***^ (.40, .42)	.39^***D***^ (.37, .40)	.73 (.73, .74)	.85 (.84, .85)	.62 (.61, .63)
Unemployed (2014)	22 (.21, .23)	.06^***E***^ (.05, .06)	.07^***E***^ (.06, .07)	.01 (.01, .01)	< .01 (.00, .00)	.04 (.03, .05)
Race/Ethnicity–White, non-Latino	51^***F***^ (.40, .52)	.59^***G***^ (.58, .60)	.48^***F***^ (.47, .50)	.62^***H***^ (.61, .62)	.62^***H***^ (.61, .63)	.59^***G*,*H***^ (.58, .61)
≥5 Office visits (within 90 days)	50^***I***^ (.49, .51)	.59 (.58, .60)	.56 (.55, .57)	.50^***I***^ (.49, .51)	.62 (.61, .63)	.66 (.64, .67)
>15 Non-face encounters						
(prior year)	69 (.68, .70)	.78^***J***^ (.77, .78)	.82^***K***^ (.81, .83)	.79^***J***^ (.78, .79)	.87 (.86, .88)	.83^***K***^ (.82, .84)
≥3 ED visits						
(prior year)	46 (.45, .47)	.42^***L***^ (.41, .43)	.41^***L*,*M***^ (.40, .42)	.39^***M***^ (.38, .40)	.38^***M*,*N***^ (.37, .39)	.36^***N***^ (.35, .38)
≥1 Inpatient admission						
(prior year)	68 (.67, .69)	.54 (.53, .55)	.64^***P***^ (.63, .66)	.64^***P***^ (.63, .64)	.65^***P***^ (.64, .66)	.57 (.56, .58)

^1^ Variables with the same superscripted letters are statistically equivalent.

## Discussion

Using Mixture Distribution IRT models, we identified 6 distinct patient subgroups among 68,400 Veterans who were at very high-risk for hospitalization. We found that the same medical diagnosis may represent different levels of accumulated complexity for a patient depending on which subgroup they are in (e.g., depression was prevalent in several subgroups, but reflected different degrees of complexity across those groups). Efficiency is illustrated in that, within this very high-risk sample, only a subset of 31 medical conditions provided the essential information to define 6 distinct patient groupings; this reduced to 12 salient medical conditions after modeling the subgroups. The unused medical conditions either lacked sufficient variance or failed to cluster consistently with other medical conditions to be useful in determining subgroup membership. Furthermore, MD-IRT also assigns each patient a complexity score, which takes into account the individual’s diagnoses in the context of her/his assigned subgroup. The 6 subgroups had distinct medical care utilization patterns, reinforcing the idea that they are qualitatively different patient populations.

This analysis also demonstrated that MD-IRT has potential to inform care plans for high-risk patients because it may define patient subgroups that could be clinically distinct, potentially benefitting from different types, or modes of, clinical interventions to reduce hospitalization rate and other negative health outcomes. Numerous prediction models have been used by healthcare systems to identify patients at risk for untoward events. Their clinical utility is, however, limited because simply categorizing an individual as “high-risk” does not necessarily provide specific guidance about what that individual needs next in their clinical care. Care coordination programs often have a one size fits all approach to high-risk patients, or rely on burdensome, time-intensive personal assessments to tailor their approach. MD-IRT has the potential to customize care approaches, by reducing the amount of information needed to a small subset of diagnoses using existing health system data, to indicate to teams what patients’ clinical needs might be without as extensive of an assessment.

Specifically, the models we present estimated the medical complexity for *each* medical condition in the context of other co-existing conditions. Two notable contributions of using the MD-IRT approach include being able to identify medical conditions that may be present in two groups, but have very different complexity estimates, and the occurrence of the same condition in different groups, with approximately the same complexity estimate. An example of the former property is having a diabetes diagnosis in both the “Complex Mental Health” and “Complex Diabetes” groups, yet with different complexity values (Fig 3). That is, diabetes was a marker of high patient complexity for the “Complex Mental Health” group, but much lower complexity for patients in the “Complex Diabetes” groups. An example of the latter property would be the presence of depression in both the “Complex Diabetes” and “Cardiac+Cancer” groups, and both had a similarly high complexity value (Fig 3). This indicates that patients with depression are very complex for both groups, yet, the comorbidities underlying that high level of complexity still differed between the groups: patients having depression in the “Complex Diabetes” group would typically also have hypertension, diabetes, renal failure, while a depressed patient in the “Cardiac+Cancer” group would likely have a cancer, hypertension, coronary artery disease, and congestive heart failure. This shows that even when diagnoses carry the same complexity estimates across groups, MD-IRT could reveal different types of complexity that could have different clinical implications, depending on the group. Future studies could confirm whether complexity values of persons with particular medical condition(s) confer with increased risk of poor outcomes among one group compared to others. In this case, other comorbidity indices that assign a single weight to a particular condition could fail to account for the heterogeneity in importance of specific conditions among subgroups, thus less accurately representing a patient’s true risk. If shown to reliably predict outcomes and beneficial interventions, patient and condition subgroup-specific complexity scores, identified by these MD-IRT methods, could be applied to risk prediction or adjustment. In addition, these methods could also be applied to other clinical domains, for example, examining whether a group of patients with mental illness diagnoses contains distinct patient subgroups, each with different salient group-specific conditions that mark levels of some construct, such as suicide risk.

Certain limitations should be kept in mind when interpreting these findings. First, diagnostic data were derived from ICD-9 codes in the patient’s VA electronic health record and some conditions may be under-reported. Second, the data on utilization, hospital admissions, and emergency room visits, were based solely on data recorded in the VA system and did not include Veteran use of non-VA services. Even so, prior research has shown that VA patients with the greatest burden of chronic illness tend to rely upon VA substantially more than healthier individuals.[2, 3, 41] Third, due to limited information on certain diagnoses, some had to be collapsed into one overarching category, such as cancer that did not account for the various forms of the disease (e.g., breast vs. lung cancers). With more specific diagnostic information, more precise groupings might be found. Fourth, results may be slightly different if patients were retained using a less stringent person fit criterion. Finally, the sample was limited to high-risk Veterans, largely men, which constrained generalizability to other patient populations.

The aim of this study was to introduce a novel application of IRT modeling using accessible medical data to present potentially clinically useful information on subgroups of high-risk patients. These distinct patient profiles provide a framework from which further exploration of high-risk patients can begin. For example, further models performed on the subgroups incorporating other data (e.g., socioeconomic) may identify additional elements key to differentiating one group’s needs from another’s.

## Conclusion

By applying MD-IRT models to data already existing in modern healthcare systems, we were able to identify diagnostic constellations among otherwise undifferentiated high-risk patients, thus efficiently grouping patients into clinically distinct subgroups with unique markers of the degree of complexity within each group. If validated, care management approaches tailored to each group’s needs and markers of complexity may more efficiently and effectively reach complex patients at risk for poor health outcomes.

## Supporting information

S1 Table ICD-9 definitions of the 31 medical conditions used to define patient subgroups: All diagnoses assessed in 2014 based on any 1 occurrence on outpatient encounter forms and inpatient records in the prior 12 months. (DOCX)

S2 Table Coefficients of final subgroup IRT 1PL and 2PL models. (DOCX)
