# Supplementary material for: Using item response theory with health system data to identify latent groups of patients with multiple health conditions
Source: PLoS One. 2018 Nov 26;13(11):e0206915. doi: 10.1371/journal.pone.0206915 (PMC6261016; doi:10.1371/journal.pone.0206915)
Supplement: S1 Table — (DOCX) [file pone.0206915.s001.docx]

**S1. ICD-9 definitions of the 31 medical conditions used to define patient subgroups: All diagnoses assessed in 2014 based on any 1 occurrence on outpatient encounter forms and inpatient records in the prior 12 months.**

| **Medical conditions** | **ICD-9 codes** |
| --- | --- |
| Diabetes | 250.xx |
| Hypertension | 401.0, 401.1, 401.9, 402.10, 402.90, 402.xx, 404.10, 404.90, 404.xx, 405.11, 405.19, 405.91, 405.99 |
| Congestive heart failure | 402.01, 402.11, 402.91, 404.01, 404.03, 404.11,404.13,404.91, 404.93, 428.xx, 425.xx |
| Coronary artery disease | 411, 414 |
| Chronic pain | 53.1 X, 250.6X, 307.81, 307.80. 307.89, 337.20-337.29, 338.21-338.29, 339.00-339.89, 346.xx, 350.1, 350.2, 353.6, 354.XX, 355.XX, 357.2, 357.3, 357.4, 357.8, 357.9, 710.xx –719.xx, 720.xx – 724.xx, 725.xx – 739.xx, 756.10-756.13, 756.19, 780.96, 784.0x, 805.xx, 806.xx, 839.1, 839.2, 839.42, 846.xx, 847.0-847.3, 847.9, 996.4 |
| Chronic renal failure | 285.21, 403.11, 403.91, 403.xx, 404.12, 404.92, 404.xx, V45.1x, V56.x, V42.0, 585.xx, 586.xx |
| Acute renal failure | 584.9 |
| Liver disease | V42.7, 567.23, 572.2, 572.3, 572.4, 572.8, 571.9, 571.8, 571.6, 571.5, 571.3, 571.2, 571.0, 456.1, 456.0, 571.xx, 070.xx, 456.20, 456.21 |
| Chronic hepatitis | 070.22, 070.23, 070.3, 070.32, 070.33, 070.4, 070.42, 070.44, 070.49, 070.52, 070.54, 070.7, 070.70, 070.71, 571.2, 571.4, 571.40, 571.5, 571.6, 571.8, 572, 573.0, 456.0, 456.1, 456.2, 456.20, 456.21 |
| Chronic arthritis | 710.0, 710.1, 712.xx, 713.xx, 714.xx, 715.xx, 719.3x, 720.xx, 721.xx, 716.5x-716.9x, 719.4x-719.6x, 719.8x, 719.9x |
| Cardiac arrhythmias | 426.0, 426.1x, 426.8x, 426.9, 427.0x, 427.1x, 427.2x, 427.3x, 427.4x, 427.60, 427.89, 427.9x, V45.01, V53.31 |
| Clotting disorders | 289.81, 289.82 |
| Anemia | 280.xx, 281.xx, 282.xx, 283.xx, 284.xx, 285.xx |
| Peripheral vascular disease | 440.xx, 443.81, 443.89, 443.9, 557.1 |
| Chronic pulmonary disease | 491.xx, 492.xx, 493.xx, 494.xx, 495.xx, 496.xx, 500.xx, 501.xx, 502.xx, 503.xx, 504.xx, 505.xx, 506.4 |
| Cerebrovascular Disease | 430.xx, 431.xx, 432.9, 433.xx, 434.xx, 435.xx, 436.xx, 437.xx, 438.13, 438.14, 438.89 and 438.9 |
| Thyroid disorders | 243.xx-244.2, 244.8, 244.9, 240.xx, 241.xx, 242.xx, 246.8, 246.9 |
| Myoneural disorders; inflammatory and toxic neuropathy | 358.00, 358.9, 357.0, 357.7 |
| Depression | 293.83, 296.2x, 296.3x, 296.82, 296.90, 296.99, 300.4, 311.x |
| Psychosis | 295.xx, 297.xx, 298.xx |
| Anxiety | 300.00, 300.01, 300.02, 300.09, 300.23, 300.20, 300.21, 300.22 |
| Post-traumatic stress disorder | 309.81 |
| Serious mental illness (episodic and paranoid disorders) | 296.0x, 296.1x, 296.4x, 296.5x, 296.6x, 296.7x, 296.80, 296.81, 296.89, 296.20, 297.9 |
| Bipolar (without major depression) | 296.0x, 296.1x, 296.4x, 296.5x, 296.6x, 296.7x, 296.80, 296.81, 296.89 |
| Alcohol abuse | 303.01, 303.90, 303.91, 303.92, 305.00, 305.01, 305.02 |
| Drug abuse | 304.xx EXCEPT (304.03, 304.13, 304.23, 304.33, 304.43, 304.53, 304.63, 304.73, 304.83, 304.93); 305.xx EXCEPT (305.03, 305.1, 305.23, 305.33, 305.43, 305.53, 305.63, 305.73, 305.83, 305.93) |
| Nicotine abuse | 305.1 |
| Malignant tumor – including recurrence | 140.xx-172.xx, 174.xx-199.xx, 200.xx-208.xx, 209.0-209.3 |
| Malignant neoplasms – non-relapse | 150.x-152.x, 155.x-158.x, 160.8, 162.x, 163.x, 174.9, 185, 196.x, 197.x, 198.xx, 199.0, 202.80, (non-relapse only: 203.0-207.0), 208.0, 208.00, 208.01 |
| Weight loss | 260.xx-263.xx, 783.21 |
| Fluid and electrolyte disorders | 276.xx |
