# Supplementary material for: Using item response theory with health system data to identify latent groups of patients with multiple health conditions
Source: PLoS One. 2018 Nov 26;13(11):e0206915. doi: 10.1371/journal.pone.0206915 (PMC6261016; doi:10.1371/journal.pone.0206915)
Supplement: S2 Table — (DOCX) [file pone.0206915.s002.docx]

S2 Table. Coefficients of final subgroup IRT 1PL and 2PL models.

| Subgroup | Discrimination, *d* | | Difficulty, *b* | |
| --- | --- | --- | --- | --- |
|  | *d* (SE) | (95%CI) | *b* (SE) | (95%CI) |
| **Substance Use** | 2.04 (0.04) | (1.97, 2.12) |  |  |
| Drug Abuse |  |  | -1.50 (0.03) | (-1.55, -1.45) |
| Depression |  |  | -0.58 '(0.02) | (-0.61, -0.54) |
| Anxiety |  |  | 0.58 (0.02) | (0.54, 0.62) |
| Liver Disease |  |  | 1.86 (0.03) | (1.80, 1.92) |
| Chronic Hepatitis |  |  | 2.37 (0.04) | (2.29, 2.45) |
| **Complex Mental Health** |  |  |  |  |
| Depression | 4.01 (0.27) | (3.47, 4.55) | -0.99 (0.02) | (-1.02, -0.95) |
| Hypertension | 1.15 (0.04) | (1.07, 1.23) | -0.78 (0.03) | (-0.83, -0.72) |
| Diabetes | 2.74 (0.20) | (2.35, 3.14) | 0.41 (0.02) | (0.38, 0.44) |
| Coronary Artery Disease | 1.41 (0.08) | (1.26, 1.57) | 1.56 (0.06) | (1.45, 1.67) |
| **Complex Diabetes** | 1.40 (0.02) | (1.36, 1.44) |  |  |
| Hypertension |  |  | -1.87 (0.03) | (-1.92, -1.82) |
| Diabetes |  |  | -0.41 (0.01) | (-0.44, -0.39) |
| Renal Failure |  |  | 0.99 (0.02) | (0.95, 1.02) |
| Depression |  |  | 2.16 (0.03) | (2.10, 2.22) |
| **Liver Disease** | 1.44 (0.05) | (1.35, 1.53) |  |  |
| Hypertension |  |  | -0.89 (0.03) | (-0.96, -0.83) |
| Diabetes |  |  | 0.34 (0.03) | (0.29, 0.39) |
| Renal Failure |  |  | 1.73 (0.05) | (1.64, 1.82) |
| **Cancer with Cardiovascular** | 1.82 (0.04) | (1.74, 1.90) |  |  |
| Hypertension |  |  | -1.36 (0.03) | (-1.41, -1.30) |
| Coronary Artery Disease |  |  | 0.37 (0.02) | (0.33, 0.41) |
| Congestive Heart Failure |  |  | 0.94 (0.02) | (0.89, 0.99) |
| Depression |  |  | 2.23 (0.04) | (2.14, 2.31) |
| **Cancer with Mental Health** | 1.69 (0.05) | (1.59, 1.78) |  |  |
| Depression |  |  | -0.51 (0.03) | (-0.57, -0.46) |
| Hypertension |  |  | -0.40 (0.03) | (-0.46, -0.35) |
| Coronary Artery Disease |  |  | 1.57 (0.04) | (1.49, 1.66) |
| Congestive Heart Failure |  |  | 2.47 (0.07) | (2.34, 2.60) |
